# A scenario for the evolution of selective egg coloration: the roles of enemy-free space, camouflage, thermoregulation and pigment limitation

**DOI:** 10.1098/rsos.150711

**Published:** 2016-04-13

**Authors:** Inmaculada Torres-Campos, Paul K. Abram, Eric Guerra-Grenier, Guy Boivin, Jacques Brodeur

**Affiliations:** 1 Instituto de Hortofruticultura Subtropical y Mediterránea ‘La Mayora’, Universidad de Málaga-Consejo Superior de Investigaciones Científicas (IHSM-UMA-CSIC), Estación Experimental La Mayora, 29750 Algarrobo-Costa, Málaga, Spain; 2 Institut de Recherche en Biologie Végétale, Département de sciences biologiques.Université de Montréal, 4101 Sherbrooke Est, Montréal, CanadaH1X 2B2; 3 Centre de Recherche et de Développement en Horticulture, Agriculture et Agroalimentaire Canada, 430 Blvd. Gouin, St-Jean-sur-Richelieu, CanadaJ3B 3E6

**Keywords:** behavioural plasticity, oviposition behaviour, egg pigmentation, *Podisus maculiventris*

## Abstract

Behavioural plasticity can drive the evolution of new traits in animals. In oviparous species, plasticity in oviposition behaviour could promote the evolution of new egg traits by exposing them to different selective pressures in novel oviposition sites. Individual females of the predatory stink bug *Podisus maculiventris* are able to selectively colour their eggs depending on leaf side, laying lightly pigmented eggs on leaf undersides and more pigmented eggs, which are more resistant to ultraviolet (UV) radiation damage, on leaf tops. Here, we propose an evolutionary scenario for *P. maculiventris* egg pigmentation and its selective application. We experimentally tested the influence of several ecological factors that: (i) could have favoured a behavioural shift towards laying eggs on leaf tops and thus the evolution of a UV-protective egg pigment (i.e. exploitation of enemy-reduced space or a thermoregulatory benefit) and (ii) could have subsequently led to the evolution of selective pigment application (i.e. camouflage or costly pigment production). We found evidence that a higher predation pressure on leaf undersides could have caused a shift in oviposition effort towards leaf tops. We also found the first evidence of an insect egg pigment providing a thermoregulatory advantage. Our study contributes to an understanding of how plasticity in oviposition behaviour could shape the responses of organisms to ecological factors affecting their reproductive success, spurring the evolution of new morphological traits.

## 1. Introduction

Behaviour plays an important role in evolutionary processes. On the one hand, behavioural plasticity can buffer the rate of evolutionary change by allowing organisms to avoid environmental selective pressures by moving away from them [1]. However, an alternative view has also been advocated, wherein behaviour can set the pace at which evolution occurs [2,3]. That is, behavioural plasticity can drive the evolution of novel genetically determined traits by exposing individuals to different selective forces, when they change their way of interacting with the current environment or move to a new environment (behavioural drive) [4]. Additionally, highly plastic behaviours, such as those that play an important role in life history, are more likely to favour the evolution of new traits, since individuals must be able to respond to environmental changes directly affecting their survival and reproduction [4].

One set of behaviours that is expected to be subject to strong selective pressures is an organism's oviposition strategy, which determines when, where and how the eggs are laid. In oviparous species without post-ovipositional maternal care, eggs are vulnerable to attack by natural enemies and exposure to the elements. Because eggs are immobile, they cannot respond to changes in mortality risk over time. This set of circumstances has shaped the evolution of ‘be prepared’ strategies [5], involving the coevolution of maternal oviposition behaviour (e.g. selection of protected sites) [6–8] with egg morphology and physiology (e.g. colour, patterning, temperature/desiccation tolerance). For example, eggs of amphibian species that lay in concealed locations tend to have less melanin pigmentation than those of species that lay in open water where there are high levels of damaging ultraviolet (UV) radiation [9]. In biological systems such as these, the evolution of new physiological or morphological traits of eggs may have originally been favoured by plasticity in oviposition behaviour, exposing them to novel selective pressures.

Most plant-dwelling arthropods tend to lay their eggs on the undersides of plant leaves [10–13], where offspring are protected against abiotic risks such as wind, rain, desiccation and overheating. Leaves also act as shelters against UV radiation since they contain compounds that absorb these damaging wavelengths [14,15]. In the predatory stink bug *Podisus maculiventris* (Hemiptera: Pentatomidae), about half of egg masses are laid on the tops of leaves [10], and egg pigmentation is associated with oviposition site selection [16]. In fact, individual females of *P. maculiventris* have the ability to selectively control the pigmentation of their eggs; females lay more pigmented eggs, which are more resistant to UV radiation, on the upper surface of leaves, whereas their eggs are lightly pigmented on the undersides of leaves [16]. Other pentatomid species studied to date have non-pigmented eggs that are mostly (but not all) laid on leaf undersides [10,17], suggesting that the egg pigmentation strategy of *P. maculiventris* could have evolved from this ancestral state, operating with the existing behavioural plasticity in oviposition site selection. Thus, *P. maculiventris* egg pigmentation, and subsequently its selective application, could be the product of ‘behavioural drive’. Building on this idea, we addressed two main questions related to the evolution of selective egg coloration in *P. maculiventris*: (i) Why would it be adaptive to shift a higher proportion of oviposition effort to leaf tops? and (ii) Why would selective egg pigmentation evolve?

**Figure 1.**
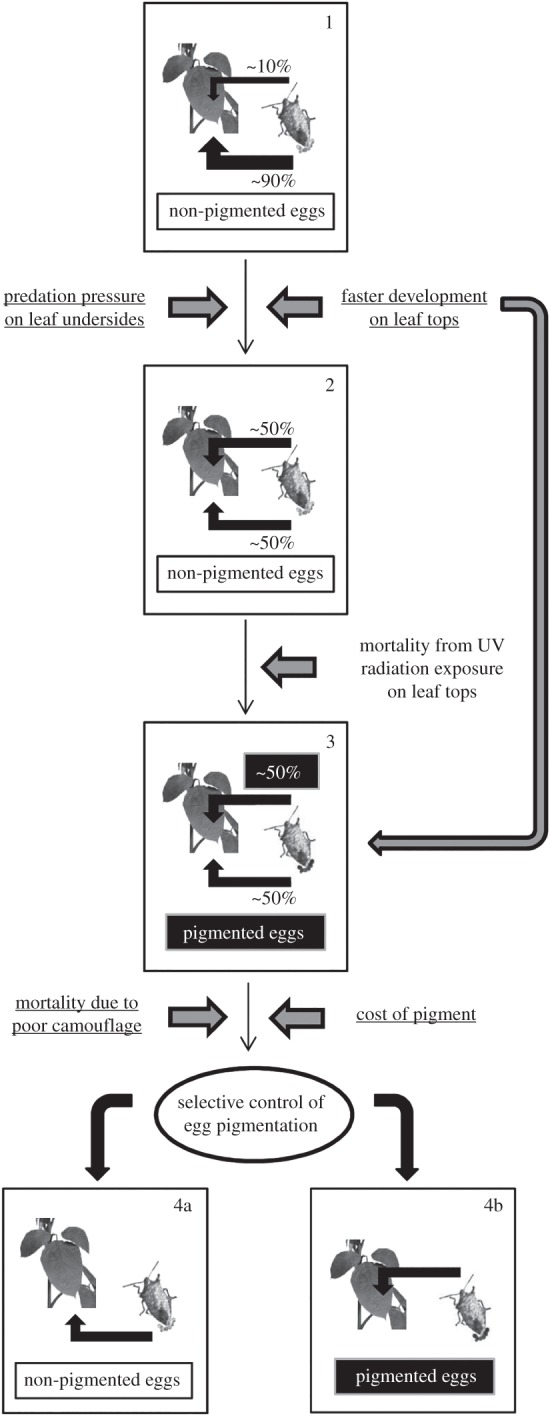
Diagram showing a hypothetical scenario for the evolution of selective control of egg pigmentation by *P. maculiventris*. In an ancestral state, stink bug females would lay non-pigmented eggs preferentially on leaf undersides (box 1). Ecological factors such as higher predation pressure on leaf undersides and/or faster egg development on leaf tops could have led stink bug females to a behavioural shift towards laying a higher proportion of egg masses on leaf tops (box 2), exposing the eggs to a new selective pressure (UV radiation damage), which then favoured the evolution of egg pigmentation (box 3). The evolution of selective control of pigment application could subsequently arise in response to selective pressures such as mortality due to poor camouflage and/or a constraint related to a physiological cost of pigment production (path from box 3 to 4a,4b). Underlined text separates hypotheses tested in the present article from those explored in other publications (non-underlined text) [16].

Regarding the first question, we tested two non-mutually exclusive hypotheses (figure 1; path from box 1 to 2):

(i) *Enemy-free space hypothesis.* The upper surface of leaves could represent ‘enemy-free’ or ‘enemy-reduced’ space [18]. Indeed, lower predation and/or parasitism pressure on the tops of leaves have been reported in other plant-dwelling arthropod systems [19–21]. Thus, if the exploitation of enemy-free space provided the impetus for the ancestors of *P. maculiventris* to shift oviposition to leaf tops, one would expect to observe overall higher rates of predation and/or parasitism of eggs on the undersides of leaves compared with leaf tops.
(ii) *Thermoregulation hypothesis*. Laying eggs on leaf tops might also confer a thermoregulatory advantage. Previous studies have indeed reported that leaf surfaces can show thermal heterogeneity [22,23] and that development times on the tops of leaves tend to be shorter [20], reducing the length of time during which individuals are susceptible to abiotic mortality factors and attack by natural enemies (i.e. the ‘slow-growth--high-mortality’ hypothesis) [24]. Thus, if surface temperatures are higher on leaf tops than on leaf undersides, this oviposition strategy could allow *P. maculiventris* eggs to accumulate more radiative heat and develop more quickly. Additionally, darker egg pigmentation could confer faster heating rates than light coloration at a given level of solar radiation, which is particularly important at low air temperatures [25,26]. If this is true, one might expect *P. maculiventris* females to fine-tune selective egg pigmentation depending on temperature, laying more pigmented eggs under cooler conditions to maximize absorption of radiative heat (*sensu* the ‘thermal melanism hypothesis’).

A shift to laying eggs on leaf tops would subsequently impose a selective pressure to evolve pigment application to protect eggs from UV radiation (figure 1; path from box 2 to 3). We next asked why selective egg pigment application evolved, whereby *P. maculiventris* females continue to lay lightly coloured eggs on the undersides of leaves. Therefore, regarding the second question, we also tested two non-mutually exclusive hypotheses (figure 1; path from box 3 to 4a,b):

(i) *Camouflage hypothesis*. One possibility is that selective egg pigmentation camouflages eggs, resulting in lower parasitism and predation rates than would be achieved by pigmenting all eggs. In oviparous animals, especially birds, a widespread adaptive strategy to prevent egg predation is the application of pigment on eggs that matches background patterning (e.g. nesting sites) with the purpose of camouflaging the eggs against predators [27,28]. Evolving an egg pigment that protects eggs on leaf tops against UV radiation could cause eggs laid on leaf undersides to be poorly matched to substrate reflectance and suffer higher rates of attack by natural enemies. Leaf undersides have a high apparent surface reflectance due to sunlight passing through them from above, whereas the surface reflectance of leaf tops is relatively low [16]. During the evolution of *P. maculiventris*' oviposition strategy, there could have been selective pressure to match the ‘brightness’ of eggs to the reflectance of oviposition substrates, reducing the contrast between eggs and leaf surfaces and providing camouflage against natural enemies. The validity of this hypothesis depends on whether the main predators and parasitoids of *P. maculiventris* eggs use visual cues, such as visual biases towards specific colours or the contrast between eggs and the oviposition substrate, to locate their prey/hosts. To provide support to the camouflage hypothesis, parasitism and predation rates would need to be higher when the colour of eggs is poorly matched to their oviposition substrate: light eggs would be attacked at a higher rate than dark eggs on leaf tops, and the reverse would be true on leaf undersides.
(ii) *Pigment cost hypothesis*. Female *P. maculiventris* may avoid applying the UV-protective pigment to eggs when it is unnecessary (i.e. on leaf undersides where eggs are already protected from UV radiation), to avoid having to pay the cost of pigment production—especially when external factors further constrain pigment synthesis. In previous studies, factors constraining pigment production such as nutrient limitation and temperature have received special attention [29,30]. Starvation reduces the overall amount of resources available to allocate to different functions, while increasing temperatures increase the rate of metabolic resource utilization. When starved or experiencing higher temperatures, metabolic resources should thus be shifted away from reproduction (including pigment production) and towards functions more critical for survival. Here, one would thus expect less pigment to be applied to eggs when *P. maculiventris* females are starved, and when they are kept at higher temperatures.

In this study, we propose an evolutionary scenario to explain the selective control of egg pigmentation of *P. maculiventris*, testing each of the proposed hypotheses and their corresponding predictions. First, we evaluated the enemy-free space, camouflage and thermoregulation hypotheses under field conditions. We tested for differences in predation/parasitism levels and embryonic development times among egg masses with different levels of pigmentation depending on leaf side (top or underside). Additionally, in laboratory experiments, we evaluated the thermoregulation and pigment cost hypotheses, determining (i) whether *P. maculiventris* egg pigmentation selectively responds to temperature, and (ii) whether the amount (modified by starvation) and rate of consumption (modified by temperature) of metabolic resources limits the amount of pigment available for eggs.

## 2. Material and methods

### 2.1. Insect colony

*Podisus maculiventris* colonies were established from individuals (approx. 200) collected from several locations in the London and Ottawa (Ontario, Canada) regions in 2011 and 2012. Colonies were maintained continuously thereafter, fed with the larvae and pupae of mealworms (*Tenebrio molitor* L.; Coleoptera: Tenebrionidae) and fresh green beans. Green polyester fabric (Fabricville, Montréal, Canada) was used as oviposition substrate. Unless stated otherwise, insects were kept at 24 ± 1°C, 50 ± 5% RH and a 16 L : 8 D photoperiod, at an illumination of 9000 ± 1000 lux, produced by linear fluorescent lights (Philips 86 W F96T8/TL841/H0/Plus).

### 2.2. Quantification of egg pigmentation

In all experiments, we quantified the degree of egg pigmentation following methods detailed elsewhere [16]. Briefly, egg brightness was measured digitally from white balance-corrected photographs of eggs against a filter paper background, under standardized lighting conditions (approx. 9000 lux). We then used a calibration curve, obtained by measuring the spectral absorbance of solubilized eggs, to convert brightness measurements to pigmentation index (PI), correcting for the nonlinearity of the digital photographs with regards to light intensity [16,31]. Resulting PI values for *P. maculiventris* can range from 0 (eggs with no pigment) to 25 (very dark eggs).

### 2.3. Enemy-free space, camouflage and thermoregulation hypotheses (field tests)

The field experiment tested for differences in predation/parasitism levels and embryonic development time among egg masses with different levels of pigmentation, depending on both leaf side (top or underside). This allowed us to test the enemy-free space, camouflage and thermoregulation hypotheses (see Introduction section). This experiment took place on soya bean plants (cultivar BeSweet, 2001 11C, Stokes, Canada) planted in peatmoss/perlite/vermiculite mix (Berger growing mix, Québec, Canada) in a large open plastic bin (l: 1.27 m, w: 0.85 m, h: 1.07 m) in a mixed field of grasses and wildflowers located in a semi-urban environment next to the Montréal Botanical Gardens (N 45 33.717, W 73 34.302). The soya bean (50 plants) was planted in late May (2014) and was in the vegetative stage (V5+, omafra.gov.on.ca) at the beginning of the experiment in mid-July, and at the full seed stage (R6) at the end of the experiment in late August. Soya bean was chosen because *P. maculiventris* and its natural enemies are often present in soya bean fields in North America [32], and part of our laboratory population of *P. maculiventris* originates from collections made in soya bean fields. Although soya bean is an introduced plant in North America and therefore not the ‘ancestral’ ecosystem for *P. maculiventris*, we assume that the ecological factors we considered with respect to leaf side (e.g. the presence of natural enemies, abiotic conditions) are generalizable to other plant systems.

Egg masses containing between 8 and 16 eggs were collected from the colony, on small pieces of green polyester fabric (1–2 cm^2^), within 24 h of being laid. Their PI was then measured as described above. Egg masses spanning the full range of pigmentation were used, and were randomly placed on either the leaf top (*n* = 70) or leaf underside (*n* = 70) of haphazardly selected plants. The pieces of fabric holding the eggs were affixed with a thin layer of waterproof, non-toxic adhesive (Gorilla Glue, Cincinnati, OH) to the uppermost set of mature trifolate leaves on each plant. After collections early in the season revealed that a large proportion of egg masses were lost (probably due to predation), we applied a ring (diameter: approx. 3 cm) of Tanglefoot glue (Grand Rapids, MI) on the leaf around a subset (*N* =  84/108) of egg masses from late July to late August, distributed between both leaf top (*n* = 42) and leaf underside (*n* = 42) treatments. We reasoned that the glue would exclude biotic (i.e. walking natural enemies during host/prey localization), but not abiotic (e.g. wind, rain) factors potentially responsible for missing eggs, allowing us to test whether missing eggs were indeed due to natural enemy attack. Accordingly, we commonly saw parasitoids (*Telenomus podisi*; Hymenoptera: Platygastridae) and predators (e.g. ladybird beetle adults and larvae, *Orius* sp.) trapped in the glue.

Egg masses were monitored for *P. maculiventris* nymph emergence twice daily, and nymphs were removed as they were counted. Eggs were left on the plants until 100 degree days (linear degree-day model with base 10.7°C; using continuous air temperature data for Montréal obtained from Environment Canada) had elapsed since they were placed in the field (see [33] for details regarding the calculation of degree days). This is 21.8 more degree days than required for the egg-nymph development of *P. maculiventris* [34], so this collection time ensured that all nymphs had emerged before eggs were removed from plants. Upon collection, eggs were brought back to the laboratory and placed in small, ventilated plastic cups (diameter: 4.2 cm, height: 1.5 cm) under standard rearing conditions to allow parasitoids to develop and emerge (approx. 10–30 days after collection; all emerging parasitoids were *T. podisi*). Following parasitoid emergence, all egg masses were dissected to score the developmental state of each egg: unemerged parasitoid, unemerged developed nymph, incomplete nymph development (aborted) or attacked by a predator.

### 2.4. Thermoregulation and pigment cost hypotheses (laboratory tests)

We conducted two laboratory experiments to test the thermoregulation and pigment cost hypotheses. The first laboratory experiment tested whether temperature affects the pigmentation of eggs laid by stink bug females. Five female and three male *P. maculiventris* (11–25 days after moulting; mean = 18.36 days) were confined to five soya bean plants, stages V1–V2, in a plastic pot (diameter: 15.2 cm, height: 10.7 cm) using a perforated plastic bread bag (27.5 × 45.0 cm) fixed to the rim of the plant pot with an elastic band. Five *T. molitor* larvae were provided as prey, confined to prevent them from escaping predation. Three replicates (i.e. plant pots) were performed at each of the three temperatures tested: 20, 25 or 30 ± 1°C. These temperatures span the linear range of the species' developmental rate curve, with 30°C being situated slightly above the thermal optimum [34]. After 72 h, egg masses were collected and their position (leaf top, leaf underside or on the plastic bag) was noted. Egg masses laid on the plastic bag were excluded from analysis. For each egg mass collected (*n* = 74), the brightness of a subset of five eggs was measured, averaged and converted to PI.

We next tested whether pigment production by *P. maculiventris* females is constrained by nutrient limitation by varying both the quantity of available resources (either starving females of insect prey or feeding them *ad libitum*) and the rate of consumption of metabolic resources (temperature). This served as a test of the pigment limitation hypothesis. Female stink bugs were collected while mating for the first time (7–10 days after moulting into adults). Following mating, females were isolated in Petri dishes (diameter: 9 cm, height: 1.5 cm) with a piece of green bean and two *T. molitor* larvae. Petri dishes were used instead of plants so that the arena size and type of plant resources could be standardized. The bottom interior surface of these Petri dishes was covered with black polyester fabric to create an oviposition substrate that would encourage females to lay more heavily pigmented eggs [16] and create conditions where pigment stores could become limited. Females were then placed at either 25.0 ± 1°C or 30.0 ± 1°C, at a photoperiod of 16 L : 8 D and 50 ± 10% RH. After the first 24 h, *T. molitor* larvae were taken away from half of the females (starved) and re-supplied daily for the other half (fed). Both feeding regimes had access to green beans, replaced every 24 h (thus, we assumed that chemical precursors for pigment production are derived from animal prey, and not plant material). Eggs were collected at least twice daily, noting their position in the arena, and the PI of all eggs was measured (instead of a subset, in case there was more variability in egg mass colour caused by pigment limitation when females were starved) in order to calculate an average brightness for each egg mass, which was then converted to PI. Experiments lasted 7 days in total, and were replicated 10 times for each of the four feeding regime/temperature combinations. A total of 135 egg masses (1457 eggs) were laid during the experiment. Cannibalism was recorded when it occurred (eggs were emptied of their contents).

### 2.5. Statistical analyses

Details regarding statistical analysis are provided in the electronic supplementary material (Methods S1). Briefly, we fitted generalized linear mixed models to temporally pseudoreplicated proportion data (emergence, recovery, parasitism, predation, abortion), accounting for overdispersion when necessary. Linear mixed models were fitted to temporally pseudoreplicated data (development time data) that met assumptions of normality and homoscedasticity. Generalized linear models were fitted to data with poisson (for count data) or quasi-binomial (for overdispersed proportion data) error distributions. All statistical analyses were conducted with the R software package, v. 2.15.1 [35].

## 3. Results

### 3.1 Enemy-free space, camouflage and thermoregulation hypotheses (field tests)

**Figure 2.**
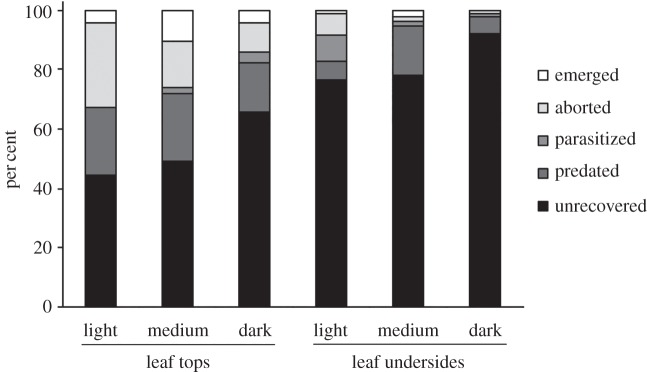
Pooled percentages of *Podisus maculiventris* eggs that emerged, aborted, were parasitized, were predated or were unrecovered, for egg masses of different colours (light, PI < 7; medium, 7 < PI < 14; dark, PI > 14) placed on leaf tops or leaf undersides. Note that the effect of egg colour was analysed as a continuous factor in subsequent statistical tests (table 1) but is presented categorically in the interest of clarity. Data are presented only for egg masses without glue applied around them (total *n* = 56 egg masses/719 eggs).

**Figure 3.**
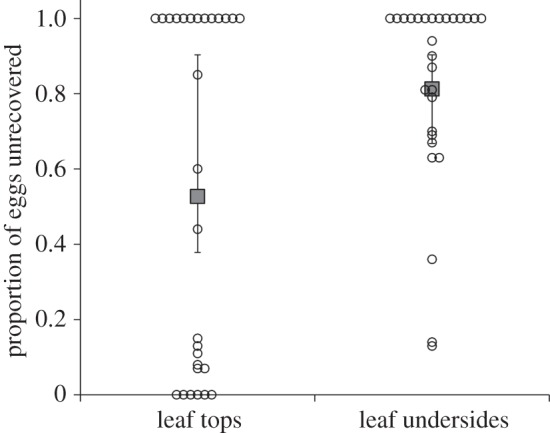
Proportion of *Podisus maculiventris* eggs that were unrecovered in the field experiment, depending on leaf side (top or underside). Each data point represents a single egg mass; points with the same value are displaced horizontally for clarity. Large, filled grey symbols are predictions (±95% CI; fixed effects only) from a generalized linear mixed model with sampling date and egg mass ID as random effects. See table 1 and text of Results for statistical information.

The overall rate of emergence and the loss of *P. maculiventris* eggs to the various mortality factors measured in the field experiment are shown in figure 2. Overall emergence rate was not significantly affected by leaf side or egg mass PI, although there was a tendency (*p* = 0.061) towards a higher emergence rate on leaf tops (table 1). Neither the proportion of parasitism nor the proportion of eggs showing direct evidence of predation was affected by leaf side or egg mass PI (figure 2 and table 1). However, the proportion of eggs that were unrecovered (probably due to removal by predators; see below) was influenced by leaf side: significantly more eggs were lost on leaf undersides than on leaf tops (figure 3 and table 1). The proportion of unrecovered eggs was not affected by egg mass PI.

**Table 1.** Statistical significance of leaf side (top versus underside), egg mass pigmentation index (PI), and their interaction for various mortality factors and development time of *Podisus maculiventris* eggs. See text of Results and figures 3–5 for effect sizes.

measurement	factor	$\chi^{2}$	*p*-value
% overall survival^a^	leaf side	3.59	0.061
	PI	0.20	0.65
	leaf side × PI	0.01	0.53
% unrecovered eggs^a^	leaf side	13.41	<0.001
	PI	1.68	0.19
	leaf side × PI	0.15	0.70
% emergence of recovered eggs^a^	leaf side	0.0176	0.89
	PI	0.58	0.44
	leaf side × PI	0.76	0.38
% parasitism^a^	leaf side	0.21	0.64
	PI	0.04	0.84
	leaf side × PI	0.01	0.92
% predation^a^	leaf side	1.51	0.21
	PI	0.59	0.81
	leaf side × PI	0.53	0.47
development time^b^	leaf side	1.25	0.26
	PI	7.31	0.0068
	leaf side × PI	3.01	0.082

^a^Likelihood ratio tests (LRTs) comparing nested generalized linear mixed models (binomial error distribution).

^b^LRTs comparing nested linear mixed models.

**Figure 4.**
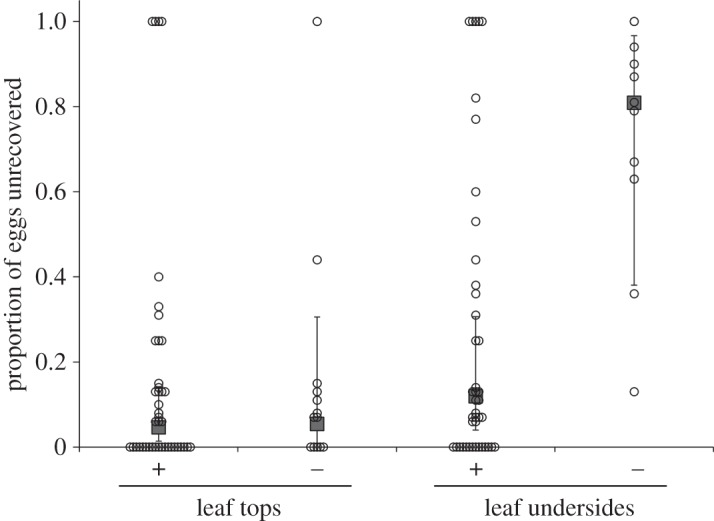
Proportion of *Podisus maculiventris* eggs that were unrecovered in the field experiment (during the period of time where glue was applied around a subset of egg masses to partially exclude natural enemies), depending on leaf side (top or underside) and whether or not glue was applied (+, applied; −, not applied). Each data point represents a single egg mass; points with the same value are displaced horizontally for clarity. Large, filled grey symbols are predictions (±95% CI; fixed effects only) from a generalized linear mixed model with sampling date and egg mass ID as random effects. See text of Results for statistical information.

Applying a ring of glue around egg masses resulted in an almost complete (97.7%) reduction in the proportion of eggs showing direct evidence of predation (*χ*^2^ = 10.76, *p* = 0.0010). The effect of glue application on the proportion of unrecovered egg masses depended on leaf side (significant position × glue application interaction; *χ*^2^ = 7.88, *p* = 0.0050; figure 4). Applying glue around eggs reduced the proportion of unrecovered eggs on the undersides of leaves (*χ*^2^ =  8.02, *p* = 0.0046), but not on the tops of leaves (*χ*^2^ = 0.0189, *p* = 0.89).

**Figure 5.**
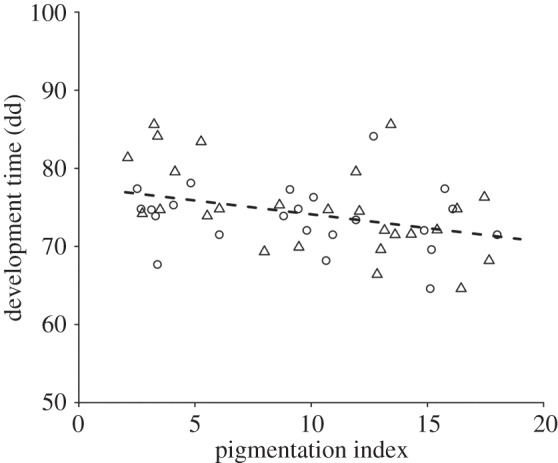
Embryonic development time, in degree days (dd), of *Podisus maculiventris* egg masses of different pigmentation levels (pigmentation index, PI) placed on soya bean leaf tops (triangular symbols) or undersides (circular symbols). The regression line of development time versus PI is derived from linear mixed model parameter estimates (fixed effects only; refer to table 1 for statistical significance).

The mean development time of *P. maculiventris* embryos expressed as degree days was influenced by egg mass PI (table 1), but it was unaffected by leaf side. Darker-coloured eggs developed faster for a given number of accumulated air temperature units (figure 5).

### 3.2. Thermoregulation and pigment cost hypotheses (laboratory tests)

**Figure 6.**
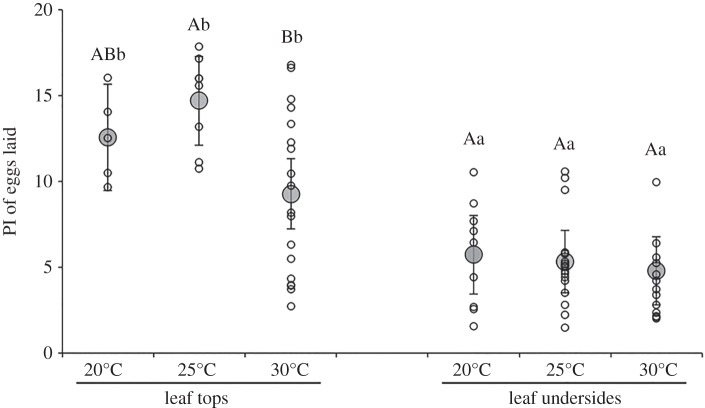
Pigmentation index (PI) of eggs laid on the tops and undersides of soya bean leaves by *Podisus maculiventris* females in the laboratory, depending on temperature. Each point represents a single egg mass. Large, filled circular symbols are predictions (±95% CI; fixed effects only) from a linear mixed model with experimental block as a random effect (see text of Results for statistical significance of each factor). Letters above bars are from Tukey contrasts (lower case letters: contrasts within temperatures between laying positions; upper case letters: contrasts within positions among temperatures). Model predictions not labelled with the same letter of a given case are significantly different (*p* < 0.05).

The PI of egg masses laid by *P. maculiventris* on soya bean plants was affected by an interaction between laying side and temperature (likelihood ratio test (LRT), *χ*^2^ = 7.15, *p* = 0.028; figure 6). Eggs were more pigmented on leaf tops than leaf undersides at all three temperatures, with between-position differences in PI ranging from 175% at 25°C down to 94% at 30°C. On leaf tops, females laid less pigmented eggs at 30°C, whereas egg pigmentation was unaffected by temperature on leaf undersides (figure 6).

In the starvation experiment, the pigmentation of egg masses was not different between fed and starved *P. maculiventris* females (table 2). However, egg masses were overall less pigmented at 30°C than at 25°C (table 2), in line with the results from the previous experiment (figure 6). Within-egg mass variation in PI was not affected by starvation treatment (LRT, *χ*^2^ = 0.68, *p* = 0.41), temperature (*χ*^2^ = 3.25, *p* = 0.071) or the interaction of these two factors (*χ*^2^ = 0.58, *p* = 0.45). Females laid similar total numbers of egg masses across treatments, but laid on average 79% more eggs when fed (i.e. there were more eggs in each mass), independent of temperature (table 2). The proportion of eggs cannibalized was increased significantly by starvation, and cannibalism was less prevalent at 30°C than at 25°C (table 2).

**Table 2.** Mean (±s.e.) egg mass pigmentation index (PI), total number of egg masses laid, total number of eggs laid, and the proportion of eggs cannibalized by *P. maculiventris* females either fed ad libitum (F) or starved (S) with respect to insect prey, and kept at one of two constant temperatures.

		temperature treatment		statistical significance		
variable	starvation treatment	25°C	30°C	starvation	temperature	starv × temp
egg mass PI^a^	F	14.73 ± 0.75	8.93 ± 0.72	*χ*^2^ = 0.01	*χ*^2^ = 15.75	*χ*^2 ^= 0.027
				*p* = 0.99	*p* < 0.001	*p* = 0.87
	S	14.79 ± 0.71	9.25 ± 1.04			
no. total egg masses^b^	F	4.80 ± 0.61	2.57 ± 0.86	$\chi^{2}=1.74$	$\chi^{2}=2.36$	$\chi^{2}=0.25$
				*p* = 0.19	*p* = 0.12	*p* = 0.61
	S	1.85 ± 0.59	1.14 ± 0.51			
no. total eggs^b^	F	54.50 ± 8.77	52.67 ± 12.82	$\chi^{2}=121.22$	$\chi^{2}=0.17$	$\chi^{2}=0.14$
				*p* < 0.001	*p* = 0.68	*p* = 0.71
	S	29.10 ± 6.62	29.40 ± 8.08			
% eggs cannabalized^c^	F	0.40 ± 0.28	0.00 ± 0.00	*F* = 128.93	*F* = 17.69	*F* = 0.25
				*p* < 0.001	*p* < 0.001	*p* = 0.62
	S	48.00 ± 8.30	15.00 ± 8.40			

^a^LRTs comparing linear mixed model with replicate (individual), number of days since the start of the experiment, and laying position as random factors. Means are pooled with respect to random factors; only significance of fixed factors is presented.

^b^LRTs comparing nested generalized linear models with poisson error structure.

^c^F-tests comparing generalized linear models with quasi-binomial error structure.

## 4. Discussion

The goal of our study was to investigate a plausible evolutionary scenario for the egg coloration strategy of the predatory stink bug *P. maculiventris*, whereby behavioural plasticity in ancestral oviposition site selection could have driven the evolution of selective pigment application to eggs. We addressed two main questions: first, what pressure(s) would have selected for proportionally more eggs being laid on leaf tops (and thus spur the evolution of UV-protective egg pigmentation); and second, what pressures would have favoured the subsequent evolution of selective egg pigmentation. Our results support the idea that behavioural plasticity in oviposition site selection could have allowed a shift towards laying eggs in ‘enemy-reduced’ space on leaf tops, then exposing the eggs to a new selective pressure—UV radiation—which would in turn select for a protective pigment. However, why *P. maculiventris* continues to lay light eggs on the undersides of leaves remains unclear, as our hypotheses to explain the evolution of selectivity in egg received weak (camouflage hypothesis) or indirect (cost of pigment production hypothesis) support.

We suggested that the upper surface of leaves could represent a safer site for eggs with lower levels of predation and/or parasitism (enemy-reduced space). Our results indicated that the proportion of eggs that were parasitized, or showed direct evidence of predation, did not depend on the position of the eggs on leaves. However, the proportion of unrecovered eggs, the most important mortality factor in our study, was overall higher for eggs placed on the undersides of leaves, and was reduced on the undersides of leaves when glue was applied in a ring around eggs. A probable explanation is that a large proportion of unrecovered eggs were removed by predators without leaving direct evidence of predation. The most likely predator is the ladybird beetle *Harmonia axyridis* (Coleoptera: Coccinellidae), which was commonly observed during the field experiment. Indeed, in preliminary follow-up laboratory experiments, most *P. maculiventris* eggs attacked by adults and larvae of *H. axyridis* were completely removed from the substrate (P. K. Abram 2014, unpublished data). Furthermore, ladybird beetles are known as important egg predators of other stink bug species [36–38]. *Harmonia axyridis* probably focuses the majority of its foraging efforts on leaf undersides, attracted over short distances by the odour of its most common prey (e.g. aphids) [39], which are typically aggregated there. Thus, the eggs of other potential prey such as *P. maculiventris* would be at a greater risk of incidental intraguild predation. Greater predation risk on leaf undersides has previously been observed for aphid mummies and plant-feeding mites [20,21], and could be a general phenomenon mediating the distribution of insects throughout plant architecture.

We hypothesized that laying eggs on leaf tops also has a thermoregulatory advantage, if surface temperatures are higher on the tops of leaves than on the undersides of leaves. Additionally, dark pigmentation could further accelerate development by allowing eggs to collect and retain more radiative heat [25,26]. We found little support for the first hypothesis but did find good evidence for the latter. Temperature tends to be similar between leaf tops and leaf undersides (I. Torres-Campos and P. K. Abram 2014, unpublished data), and, correspondingly, there was no difference in the development time of eggs between leaf tops and leaf undersides. However, development time decreased with increasing levels of egg pigmentation. To the best of our knowledge, this is the first evidence of an insect egg pigment providing a thermoregulatory benefit. Rapid development mediated by dark coloration has, however, been observed in other life stages for several other arthropod species; for example, in the cabbage moth, high densities of larvae induce a switch towards dark phase larvae to develop faster and avoid competition for food [40]. In addition, we found that in the laboratory, stink bugs did tend to lay more pigmented eggs at the two lower temperatures tested, similar to what is predicted by the thermal melanism hypothesis [41], in that dark coloration provides faster heating rates than light coloration in cooler environments. Although we cannot quantify whether differences in egg pigmentation among temperatures would be large enough to confer a significant advantage in terms of development time, it seems more likely that this result could have instead been due to a constraint on pigment production at higher temperatures (see below). Overall, our results suggest that laying a greater proportion of eggs on the tops of leaves probably did not evolve as a result of a thermoregulatory selective advantage; however, once the pigment evolved, it may have provided an additional benefit by allowing eggs to develop faster. It is also possible the pigment could play an additional role in protection against desiccation of eggs, reducing water loss or increasing dehydration tolerance at dry conditions; body melanization has been shown to play this role in other insect species [42–44].

Assuming that shifting a greater proportion of oviposition effort on leaf tops spurred the evolution of egg pigmentation in *P. maculiventris*, what factor(s) would subsequently select for discriminating application of the pigment (i.e. not applying it when egg masses are laid on leaf undersides)? Perhaps the most attractive explanation is that matching egg colour to substrate reflectance camouflages eggs from predators and parasitoids. We thus predicted that mortality from natural enemies would increase with increasing egg pigmentation on leaf undersides (which have a high surface reflectance due to sunlight passing through them), and the opposite trend on leaf tops (which have a relatively low surface reflectance). Our results did not match these predictions. Neither the egg mass coloration itself nor its interaction with leaf side affected the proportion of eggs that were predated, parasitized or unrecovered. It is possible that other cues (e.g. infochemicals) play a more important role than visual cues associated with colour contrast during host/prey localization by natural enemies of stink bug eggs [39,45,46].

The evolution of reproductive strategies is often shaped by ecological and physiological constraints. Thereby, we hypothesized that *P. maculiventris*' strategy to preferentially apply pigment to eggs only when there is a high risk of UV exposure (i.e. when eggs are laid on leaf tops) [16] would minimize the physiological cost of pigment production. If pigment production is costly [47,48], we would expect females to lay less pigmented eggs when subjected to metabolically stressful conditions (starvation, high temperatures). Overall, this hypothesis was partially supported by our data. The mean pigmentation level of eggs laid by starved females was not significantly different from those fed ad libitum, despite the fact that females deprived of food showed clear evidence of starvation; there were higher levels of filial cannibalism by starved females, and they laid fewer eggs per egg mass. Although starvation did not affect egg pigmentation levels, we cannot rule out the possibility that pigment production is costly—since starved females laid fewer eggs, an effect of nutrient limitation on pigment supply could have been masked by a reduction in the number of eggs laid (i.e. there was less pigment available, but also less eggs on which pigment had to be applied). We did observe that females laid overall less coloured eggs at above-optimal, stressful temperatures (30°C) in two of our experiments. These results support our hypothesis that stressful temperatures could cause a shift in metabolic resource allocation away from pigment production and towards survival (somatic maintenance). However, we still cannot rule out that changes in egg pigmentation at different temperatures are actually adaptive, providing thermoregulatory benefits (*sensu* the ‘thermal melanism hypothesis’, see above); or alternatively that reducing dark pigmentation at high temperatures (30°C) could provide protection for embryos against excessive heat stress.

In summary, we have provided some evidence in support of the hypothesis that ancestral plasticity in *P. maculiventris* oviposition site selection behaviour allowing this species to shift its oviposition efforts from the underside to upper surface of leaves, could have driven the evolution of selective control of egg pigmentation (figure 1). Future work should investigate how females make the choice of where to lay their egg masses under natural conditions, based on factors such as predation risk and nutritional status. For example, *P. maculiventris* may be able to detect cues from potential egg predators [49,50], and use this information to dynamically shift more oviposition effort to the tops of leaves. This biological model system could prove to be ideal for the study of how animal egg laying strategies evolved in response to biotic and abiotic factors, and how behavioural plasticity can spur the evolution of novel morphological traits, and as a consequence the exploitation of a wider range of habitats. Although these issues remain to be thoroughly explored, they may be relevant to the reproductive strategies of many animal species.

## Supplementary Material

ESM. Methods S1. Detailed information regarding statistical analyses

## Data accessibility

Datasets for all experiments reported in this paper are publicly accessible in the Dryad data repository at http://dx.doi.org/10.5061/dryad.5qt2k.
