# Supplementary material for: A scenario for the evolution of selective egg coloration: the roles of enemy-free space, camouflage, thermoregulation and pigment limitation
Source: R Soc Open Sci. 2016 Apr 13;3(4):150711. doi: 10.1098/rsos.150711 (PMC4852638; doi:10.1098/rsos.150711)
Supplement: ESM. Methods S1. Detailed information regarding statistical analyses [file rsos150711supp1.docx]

*Methods S1 – Details of statistical analyses*

*Field tests*

We ran a series of statistical models to determine the effects leaf side and egg pigmentation (PI) on the proportion of eggs that (i) successfully emerged, (ii) were not recovered, (iii) were parasitized, (iv) showed direct evidence of predation, or (v) were neither parasitized nor predated, but did not emerge. The first two proportions were calculated with all eggs in the mass as the denominator, while the latter three proportions were calculated with only the number of recovered eggs as the denominator. These analyses were performed over the entire experimental period, and only for egg masses that did not have glue applied around them (n = 56). Statistical significance was assessed with generalized linear mixed models (GLMMs; [1]) with a binomial error distribution. Setup date (the day egg masses were placed in the field) was included as a random factor to account for temporal variability and to account for a lack of balance in the experimental design (i.e., different numbers of replicates performed for each setup day and some egg masses were not recovered). To account for over-dispersion, we also included observation-level random effects in these models [2,3]. The significance of fixed factors in each model was determined using likelihood ratio tests (LRTs) comparing simplified models with and without the factor in question [4] also testing for interactions between fixed factors.

To examine the effect of glue application on the various mortality factors, similar analyses were performed, but using only data for the time period during which the glue was applied to a subset of egg masses (total N= 108), and including glue application (present or absent) as an additional factor in the analyses. The effect of glue application on parasitism was not tested, since the incidence of parasitism was too low during this period to ascertain significance (only two egg masses were parasitized).

The effect of leaf side and PI on the mean time of embryonic development of each egg mass (in degree days; see above) was tested using a linear mixed model with the same random factors listed above, after verifying assumptions of model error normality and homoscedasticity. Egg masses with and without glue application were pooled for this analysis, after verifying that the glue did not have a significant effect on development time (LRT, χ^2^_1_ = 2.81, p = 0.094). All statistical analyses were conducted with R software package, version 2.15.1 [5].

*Laboratory tests*

For the first laboratory experiment testing the effect of temperature on egg mass PI, we ran a linear mixed model with temperature and laying position as fixed factors and replicate (plant pot) as a random factor.

For the starvation experiment, with egg mass PI as the dependent factor, we again used a linear mixed model including starvation treatment, and temperature treatment as fixed factors, and replicate (stink bug individual), the number of days since the start of the experiment, and laying position (top, side, or bottom of inner surface of petri dish) as random factors. Using a GLMM (Gamma error distribution) with the same fixed and random factors, we also tested whether the within-egg mass coefficient of variation of PI varied significantly among treatments (i.e., whether high temperature or starvation caused higher variability in within-egg mass pigmentation). Finally, for each stink bug female, we tested the dependence of the total number of egg masses laid (GLM with Poisson error structure) the number of total eggs laid (GLM with Poisson error structure), and the proportion of eggs cannibalized (GLM with quasi-binomial error structure) on starvation and temperature treatment.

For all analyses, significance of each factor was determined using LRTs, as described above (except in the case of linear models and GLMs using quasi-likelihood, in which case F-tests were used). Residual plots were used to confirm adequate model fit. Predictions and 95 % confidence intervals (CI) for each model were obtained using the lsmeans package in R.

*References*

1. Bolker B, Brooks M, Clark C, Geange S, Poulsen J, Geange SW, White JS. 2009 Generalized linear mixed models: a practical guide for ecology and evolution. *Trends Ecol. Evol.* **24**, 127-135.(doi: 10.1016/j.tree.2008.10.008)
2. Elston D, Moss R, Boulinier T, Arrowsmith C, Lambin X. 2001 Analysis of aggregation, a worked example: numbers of ticks on red grouse chicks. *Parasitol.* **122**, 563-569. (doi:10.1017/S0031182001007740)
3. Harrison XA. 2014 Using observation-level random effects to model overdispersion in count data in ecology and evolution. *PeerJ* **2**, e616. (doi: 10.7717/peerj.616)
4. Crawley MJ. 2007 *The R book*. John Wiley & Sons, UK.
5. R Core Team. 2013 R: A language and environment for statistical computing. Vienna, Austria: R Foundation for statistical computing
